# Chemical composition and standardized ileal digestibility of crude protein and amino acid in whole yeast and autolyzed yeast derived from sugarcane ethanol production fed to growing pigs

**DOI:** 10.5713/ab.21.0540

**Published:** 2022-04-30

**Authors:** Chanwit Kaewtapee, Nontawut Jantra, Krittaya Petchpoung, Choawit Rakangthong, Chaiyapoom Bunchasak

**Affiliations:** 1 Department of Animal Science, Faculty of Agriculture, Kasetsart University, Chatuchak, Bangkok, 10900, Thailand; 2 Scientific Equipment and Research Division Kasetsart University, Bangkhen Campus, Bangkok 10900, Thailand

**Keywords:** Amino acid, Autolysis, Digestibility, Ethanol, Pig, Yeast

## Abstract

**Objective:**

This research determined the chemical composition and the apparent and standardized ileal digestibility (AID and SID) of crude protein (CP) and amino acids (AA) in whole yeast and autolyzed yeast derived from sugarcane ethanol production fed to growing pigs.

**Methods:**

Six growing pigs were randomly allocated in a replicated 3×3 Latin square design with 3 diets and 3 periods of 7 days each, resulting in a total of 6 experimental replications. Three assay diets were formulated using whole yeast, autolyzed yeast, or soybean meal as the sole sources of dietary CP and AA. Pigs were allowed to adapt to the assay diets for 5 days. Thereafter, ileal digesta samples were collected continuously for 8 hours on days 6 and 7.

**Results:**

There was no difference in the chemical composition between whole yeast and autolyzed yeast, but whole yeast had low digestibility of CP and AA due to the presence of a rigid cell wall. As conducting autolysis can induce cell wall damage, the AID and SID of CP and AA were greater in autolyzed yeast than in whole yeast.

**Conclusion:**

The information obtained on the SID of CP and AA in both yeast products can be used for the accurate estimation of the bioavailability of CP and AA in feed formulations. The yeast products derived from sugarcane ethanol production are an alternative protein source in pig diets.

## INTRODUCTION

Thailand is one of the top-five countries for sugarcane production globally with annual exports of approximately 10 million metric tons, ranking second behind Brazil [1]. Sugarcane molasses is a by-product of sugar production and is widely used as a substrate for ethanol fermentation [2]. This process is commonly performed using *Saccharomyces cerevisiae* as this yeast strain has high potential for fermenting sugar to produce a high ethanol concentration [3]. As the yeast biomass is a rich source of nutritional values, including protein, peptides, amino acids (AA), carbohydrate, fatty acids, vitamins and minerals [4], the sugarcane yeast derived from ethanol production can be included in animal feed as an alternative protein source [5].

Whole yeast typically consists of the cell wall, periplasm, plasma membrane, organelles and cytoplasm [6]. Nutritionally, the highly digestible compounds (including proteins, AA, polysaccharides and nucleotides) are present in intracellular constituents [7], whereas the major structural components strengthening the yeast cell wall are glucans, mannans and chitin [6]. The rigid yeast cell wall has limited digestibility due to the absence of those endogenous enzymes in the small intestine [8]. Alternatively, the autolysis of yeast cell disruption has been reported using mechanical (bead mill, high pressure homogenizer, and ultrasound) and non-mechanical (electrical, enzymatic, physical such as osmotic pressure or heat shock, and chemical) methods to release intracellular bio-active compounds [7].

Numerous yeast products are commercially produced and supplemented in animal feed to improve growth performance and gut health function [4]. As standardized ileal digestibility (SID) of crude protein (CP) and AA has been widely accepted as a more suitable approach for bioavailability of CP and AA in feed ingredients [9], the SID of CP and AA have been reported for brewer’s yeast [10], torula yeast [11], yeast extract [12,13] and yeast products produced by the ethanol industry [14]. However, there is no information on the nutrient digestibility of yeast products derived from sugarcane ethanol production. The additional process of autolysis method may break the rigid yeast cell walls, and then release intracellular nutrients, resulting in an improvement of protein digestion in growing pigs. Therefore, the objective of this research was to determine the chemical composition, apparent ileal digestibility (AID) and SID of CP and AA in whole yeast and autolyzed yeast fed to growing pigs. For comparison, the chemical composition and the AID and SID of CP and AA were determined in soybean meal, as it is a protein-rich ingredient used extensively in swine diets.

## MATERIALS AND METHODS

The research proposal was reviewed and approved by the Animal Care and Use for Scientific Research Committee, Kasetsart University, Bangkok, Thailand (ACKU64-AGR-021) and care of animals throughout this experiment was in an accordance with the corresponding Ethical Principles for the Use of Animals for Scientific Purposes [15].

### Animals, housing, and surgical procedures

Six growing pigs (Duroc×Large white×Landrace) with initial mean (±standard deviation) body weight (BW) of 67.60 ±6.15 kg were used to determine AID and SID for CP and AA in whole yeast, autolyzed yeast, and soybean meal. The pigs were housed in individual pens (2.0×2.5 m). An evaporative cooling system was used to control environmental temperature with an average of 25°C to 26°C. Each pen was equipped with a low-pressure drinking nipple that allowed free access to water. The pigs were surgically fitted with a simple T-cannula at the distal ileum according to the procedures described by Li et al [16]. Until surgery and during the recovery period, pigs were fed a commercial grower diet with 17.5% CP and 3,200 kcal metabolizable energy (ME)/kg (as-fed basis). The pigs were allowed a recuperation period of at least 7 days.

### Experimental design, diets, and procedures

Six growing pigs were randomly allocated in a replicated 3×3 Latin square design with 3 diets and 3 periods of 7 days each, resulting in a total of 6 experimental replications. Experimental diets were fed to the pigs at a daily level of 2.5 times the estimated maintenance requirement for energy (197 kcal metabolizable per BW^0.60^; NRC [17]). Body weight was determined at the beginning of each experimental period to keep the daily feed intake constant in relation to the animals’ BW throughout the experiment. The diets were divided into 2 equal meals and fed at 0800 and 1600 h.

**Figure 1:**
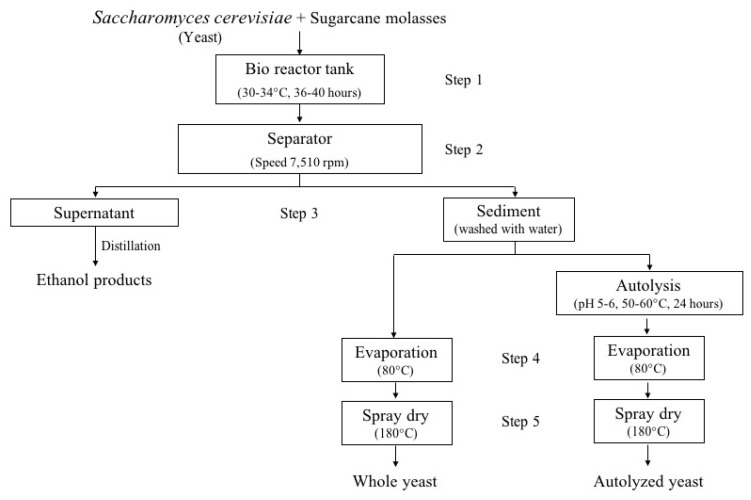
Processing steps for yeast products derived from fermentation of *Saccharomyces cerevisiae* using sugarcane molasses.

Soybean meal, whole yeast, and autolyzed yeast were used as the sole sources of CP and AA. The soybean meal was purchased from Thai Vegetable Oil Public Company Limited (Thonburi, Bangkok, Thailand) and used as the control group. The commercial yeast products, consisting of whole yeast (KOBOTEN) and autolyzed yeast (KOBOTENnutri30), were supplied by Mitr Phol Bio Fuel Co., Ltd. (Bangkok, Thailand). The processing steps for the yeast products are presented in Figure 1. In brief, *Saccharomyces cerevisiae* was used as a selected yeast strain to supplement in sugarcane molasses under fermentation conditions at 30°C to 34°C for 36 to 40 h in a bio reactor tank (Step 1). Thereafter, the yeast biomass was separated from solubles using centrifugation at 13,614 g (Step 2). The supernatant was carefully removed and used to produce ethanol, whereas the remaining sediment (yeast cells) was washed with water (Step 3). After that, the sediment was dried at 80°C under the evaporation system (Step 4). Finally, spray drying occurred at 180°C (Step 5) to prepare the powder form of whole yeast. For autolysis of the yeast product, the sediment from Step 3 was further autolyzed at pH 5 to 6 at 50°C to 60°C for 24 h, followed by Steps 4 and 5, respectively.

Three assay diets were formulated using whole yeast, autolyzed yeast, and soybean meal as the sole sources of dietary CP and AA (Table 1) to meet or exceed the dietary threshold levels for CP and AA according to NRC [17] nutrient recommendations for pigs from 50 to 75 kg BW. All diets contained 0.5% chromic oxide as an indigestible index. Vitamins and minerals were supplemented to the assay diets to meet or exceed the requirement estimate in NRC [17]. An N-free diet (Table 1) was used to determine basal ileal endogenous losses of CP and AA (IAA_end_) in an additional period at the end of the experiment.

In each of the 4 experimental periods, pigs were allowed to adapt to the assay diets for 5 days. Thereafter, ileal digesta samples were collected continuously for 8 h on days 6 and 7. The samples were collected using plastic bags attached to the barrel of the cannula with elastic bands. The bags were changed whenever they were filled with digesta and immediately frozen at −18°C. During collection, 4 mL of 2.5 M formic acid were added to the sampling bags to minimize further microbial fermentation. The frozen ileal digesta samples were allowed to thaw at room temperature, and mixed within animal and period. The ileal digesta samples were dried using freeze dryer (FDB-5503; Operon Co., Ltd., Gyeonggi-do, Korea), and were finely ground to 0.5 mm before chemical analysis.

### Chemical analyses

The samples (soybean meal, whole yeast, autolyzed yeast, assay diets and ileal digesta) were analyzed in duplicate. Official standard methods [18] were used to determine the contents of dry matter (DM; method 930.15), ether extract (method 920.38), crude fiber (method 978.10), CP (method 984.13), neutral detergent fiber (NDF; method 2002.04), and acid detergent fiber (ADF; method 973.18). The CP contents were calculated by multiplying the content of nitrogen by 6.25. The AA contents were determined using ultra high performance liquid chromatography (UHPLC) (Nexera X2; Shimadzu Scientific Instruments, Kyoto, Japan) after being hydrolyzed with 6 N HCl for 18 h at 115°C. Tryptophan was not determined. The chromium contents in the diet and ileal digesta samples were analyzed using a spectrophotometer (UV 1800; Shimadzu Scientific Instruments, Japan) according to the AOAC [18] procedure (method 968.088D).

### Calculations

The AID of CP and AA in the assay diets were calculated according to the equation:


$${\text{AID}}_{\text{D}}=[1-({\text{I}}_{\text{D}}\times {\text{A}}_{\text{I}})/({\text{A}}_{\text{D}}\times {\text{I}}_{\text{I}})]\times 100\%$$

Where AID_D_ = AID of CP and AA in assay diet (%), I_D_ = marker content in the assay diet (g/kg DM), A_I_ = CP or AA content in ileal digesta (g/kg DM), A_D_ = CP or AA content in the assay diet (g/kg DM), and I_I_ = marker content in ileal digesta (g/kg DM).

The SID of CP and AA in whole yeast, autolyzed yeast and soybean meal were calculated by correcting the AID of CP and AA in the assay diet for IAA_end_. The values for IAA_end_ were obtained using an N-free diet in combination with an indigestible marker according to the equation:


$${\text{IAA}}_{\text{end}}={\text{A}}_{\text{I}}\times ({\text{I}}_{\text{D}}/{\text{I}}_{\text{I}})$$

where IAA_end_ = IAA_end_ in g/kg DM intake.

The SID of CP and AA were calculated using the equation:


$${\text{SID}}_{\text{D}}={\text{AID}}_{\text{D}}+({\text{IAA}}_{\text{end}}/{\text{A}}_{\text{D}})\times 100\%$$

where SID_D_ = SID of CP and AA in yeast products and soybean meal.

### Statistical analysis

Homogeneity of variances and normal distribution of data were confirmed using the UNIVARIATE procedure of the SAS software package (SAS Inst., Inc., Cary, NC, USA). Outliers were identified using the MIXED procedure of SAS, where the data with studentized residuals greater than 3.0 were considered outliers and excluded from further statistical analyses. The data were subjected to mixed modeling using diet as a fixed effect and pig and period as random effects. The individual pig was the experimental unit. The significant differences between treatments based on a t-test were set at α = 0.05 using the algorithm for letter-based representation of all pair-wise comparison according to Piepho [19].

## RESULTS

The pigs remained healthy and readily consumed their assay diets throughout the experiment. Two outliers were detected for the pigs fed with soybean meal in period 2 and whole yeast in period 3. The AID and SID of CP and AA were considered outliers as influential and hence excluded from further statistical analysis. Therefore, the number of observations included in the model was 5 for whole yeast and soybean meal, and 6 for autolyzed yeast.

### Chemical composition

The analyzed chemical compositions of soybean meal, whole yeast and autolyzed yeast are shown in Table 2. The CP contents were lower in whole yeast and autolyzed yeast (341.6 and 334.3 g/kg DM, respectively) than in soybean meal (467.8 g/kg DM). The ether extract contents were lower in soybean meal (13.2 g/kg DM) than in whole yeast and autolyzed yeast (81.6 and 89.8 g/kg DM, respectively), whereas the crude fiber contents were greater in soybean meal (50.8 g/kg DM) than in both yeast products (8.8 g/kg DM). The NDF contents were greatest in whole yeast (165.0 g/kg DM) and lowest in autolyzed yeast (68.6 g/kg DM) with intermediate contents for soybean meal (137.4 g/kg DM), whereas the ADF contents were greater in soybean meal (76.8 g/kg DM) than in whole yeast and autolyzed yeast (21.7 and 2.2 g/kg DM, respectively). The ash contents were greatest in whole yeast and autolyzed yeast (244.5 and 256.0 g/kg DM, respectively) compared to soybean meal (68.5 g/kg DM). The contents of all AA samples were greatest in soybean meal, ranging for indispensable AA from 5.3 g/kg DM for methionine to 34.4 g/kg for leucine. For yeast products, the contents of indispensable AA in whole yeast ranged from 3.0 g/kg DM for histidine to 24.9 g/kg DM for arginine, whereas those values in autolyzed yeast ranged from 3.0 g/kg DM for histidine to 25.8 g/kg DM for arginine. There were relatively small differences in chemical composition and AA between whole yeast and autolyzed yeast.

### Apparent and standardized ileal CP and AA digestibility of whole yeast, autolyzed yeast, and soybean meal

The AID and SID of CP and almost all AA were lowest (p< 0.05) in whole yeast compared to those digestibilities in autolyzed yeast and soybean meal (Tables 3, 4). The AID and SID of CP in autolyzed yeast were not different from those digestibilities in soybean meal. In comparison, the AID of histidine, threonine, cystine and serine was greatest (p<0.05) in soybean meal, but the AID of alanine was greatest (p<0.05) in autolyzed yeast. The SID of threonine and serine was greater (p<0.05) in soybean meal than in autolyzed yeast, whereas the SID of alanine and glycine was greater (p<0.05) in autolyzed yeast than in soybean meal. For other AA, no differences in the AID and SID were observed.

## DISCUSSION

In the present study, the yeast products derived from sugarcane ethanol production had greater contents of fat and ash, but lower contents in CP and almost AA compared to those digestibilities in brewer’s yeast [10,17], torula’s yeast [11], single cell protein [17] and yeasts from grain ethanol co-products [20]. The variation in nutrient contents of the yeast products may have been due to the different substrates [4] and the specific yeast strains [7,11]. For example, *Saccharomyces cerevisiae* (brewer’s yeast) uses cereal grain as a substrate [21], whereas *Candida utilis* (torula’s yeast) uses wood pulp from paper manufacturing as a substrate [4,22], resulting in different chemical compositions. As shown in the tabulated values [17], the CP contents were 46.52% and 51.17% DM for brewer’s yeast and torula’s yeast, respectively. The indispensable AA ranged from 0.74 for methionine to 3.22% DM for lysine in brewer’s yeast, and from 0.64 for methionine to 3.41% DM for leucine in torula’s yeast. In the present study, sugarcane molasses was used as a substrate and fermented with *Saccharomyces cerevisiae*. The lower nutrient contents in sugarcane molasses than in cereal grain or wood pulp may have resulted in the relative low contents of CP and AA in the whole yeast and autolyzed yeast samples compared to other yeast products.

The AID and SID of CP and AA were lower in whole yeast, but for autolyzed yeast were within the range of published digestibility for brewer’s yeast [10], torula’s yeast [11], and yeast extract [12,13]. As the yeast cell has a rigid cell wall containing mainly glucans and mannans and a minor structure of chitin and protein [23], the lack of endogenous enzymes in the small intestine against these component [8] might have lowered the digestibility of CP and AA in whole yeast. Alternatively, the adjusted conditions of pH and temperature in industrial processing can cause cell autolysis that releases nutrients (such as CP and AA) from the interior of the yeast cell [7], resulting in greater AID and SID of CP and AA in autolyzed yeast than in whole yeast. The similar range of nutrient digestibility in autolyzed yeast compared to other yeast products suggested that *Saccharomyces cerevisiae* extracted from sugarcane ethanol production had good quality and was the same as brewer’s yeast, torula’s yeast and yeast extract. Notably, the AID and SID of CP and AA in autolyzed yeast were greater than those digestibility in single cell protein [17]. One possible explanation is that the single cell protein may be produced from bacterial protein containing high nucleotide contents, which are poorly digested by pigs [24].

The autolysis process can produce a small size of yeast cell debris with thinner intracellular layers [25]. This phenomenon may be more beneficial to improve nutrient digestion and absorption of autolyzed yeast for host animal. In the present study, the degradation of yeast cell wall can be observed by lower NDF and ADF contents in autolyzed yeast than in whole yeast. According to previous studies, the low NDF contents can reduce endogenous AA losses [26], and increase retention time of digesta in the gastro-intestinal tract of pigs [27]. In contrast, greater NDF and ADF contents may induce the specific endogenous CP and AA losses [28], resulting in impaired ileal digestibility of CP and AA [29]. Therefore, the small protein molecule and the relatively low fiber fraction in autolyzed yeast might be the reasons for enhancing AID and SID of CP and AA in growing pigs.

Soybean meal is widely used as a major protein source for pigs [30]. The chemical composition of brewer’s yeast is comparable to the nutrient composition of dehulled soybean meal [4]. Likewise, the AID and SID of CP and AA in brewer’s yeast [10] were within the ranges of those digestibility reported in soybean meal [17]. The AID and SID of CP and almost all the AA in soybean meal in this study were close to the tabulated digestibility [17]. Compared with soybean meal, the digestibility of CP and AA were lower in whole yeast, but were close to those digestibility for autolyzed yeast. This result suggested that autolyzed yeast can be used as an alternative protein feedstuff for inclusion in pig diets. Therefore, the reduction in soybean imports could be partly compensated by using the autolyzed yeast derived from sugarcane ethanol production.

## CONCLUSION

There were no significant differences in the chemical composition (except for NDF and ADF) between whole yeast and autolyzed yeast; however, whole yeast had low digestibility of CP and AA due to the presence of a rigid cell wall and no digestive enzymes in the pigs. Autolysis could damage the cell wall and release intracellular nutrient contents; thus, the AID and SID of CP and AA were greater in autolyzed yeast than in whole yeast. The information obtained on the SID of CP and AA in both yeast products could be used to provide accurate estimates of the bioavailability of CP and AA in feed formulations. Therefore, yeast products derived from sugarcane ethanol production are suitable as an alternative protein source for pig diets. However, further research is needed to assess the effect of dietary supplementation with autolyzed yeast on growth performance, carcass quality and the gut microbiota composition of pigs.

## Figures and Tables

**Table 1:** Composition of assay diets for determination of apparent and standardized ileal digestibility of crude protein and amino acids in whole yeast, autolyzed yeast and soybean meal (%, as-fed basis)

Item	Assay diet	N-free
Test product	47.86	-
Corn starch^1)^	36.42	66.28
Dextrose^2)^	9.00	20.00
Cellulose^3)^	2.00	5.00
Soybean oil^4)^	2.00	4.00
Calcium carbonate	0.99	-
Monodicalcium phosphate^5)^	0.60	3.00
Vitamin and mineral premix^6)^	0.30	0.30
Sodium chloride	0.30	0.40
Vitamin E^7)^	0.03	0.02
Magnesium oxide^8)^	-	0.10
Potassium carbonate^9)^	-	0.40
Chromic oxide	0.50	0.50

1) Xingmao, Zhucheng Xingmao Corn Developing Co. Ltd., Weifang, China.

2) Fufeng, Fufeng Group, Beijing, China.

3) Arbocel, J. Rettenmaier & Söhne GmbH+Co.KG, Rosenberg, Germany.

4) Thai Vegetable Oil Public Company Limited, Thonburi, Bangkok.

5) Chanhen, Guizhou Chanhen Chemical Corporation, Fuquan, China.

6) Wintermix pig finisher, Vitec Formulation Co., Ltd, Samut Sakhon, Thailand; provided the following quantities and vitamins per kilogram of assay diet: vitamin A, 1 MIU; vitamin D_3_, 0.2 MIU; vitamin E, 2 g; vitamin K_3_, 0.2 g; vitamin B_1_, 0.2 g; vitamin B_2_, 0.5 g; vitamin B_6_, 0.2 g; vitamin B_12_, 0.0024 g; nicotinic acid, 1.5 g; pantothenic acid, 1 g; folic acid, 0.1 g; biotin, 0.01 g; choline chloride, 20 g; Fe, 28 g (FeSO_4_·H_2_O); Cu, 24 g (CuSO_4_·5H_2_O); Mn, 8 g (MnO); Zn, 20 g (ZnO); Co, 0.2 g (2CoCO_3_·3Co(OH)_2_·H_2_O); I, 0.2 g (Ca(IO_3_)_2_); Se, 0.02 g (Na_2_SeO_3_).

7) TOCOMIX 500, Impextraco, Belgium.

8) Qrëc, Qrec Chemicals Co. Ltd., Selangor, Malaysia.

9) Kemaus, Elago Enterprises Pty Ltd., Sydney, NSW, Australia.

**Table 2:** Chemical composition and amino acid contents of whole yeast and autolyzed yeast (g/kg dry matter)

Item	Soybean meal	Whole yeast	Autolyzed yeast
Dry matter	909.8	958.0	953.1
Crude protein	467.8	341.6	334.3
Ether extract	13.2	81.6	89.8
Crude fiber	50.8	8.8	8.8
Neutral detergent fiber	137.4	165.0	68.6
Acid detergent fiber	76.8	21.7	2.2
Ash	68.5	244.5	256.0
Indispensable amino acids			
Arginine	32.6	24.9	25.8
Histidine	5.9	3.0	3.0
Isoleucine	18.3	11.6	12.1
Leucine	34.4	20.9	20.6
Lysine	27.5	14.8	15.3
Methionine	5.3	4.7	4.6
Phenylalanine	22.6	11.3	11.2
Threonine	18.2	15.2	15.3
Valine	17.1	9.2	9.2
Dispensable amino acids			
Alanine	20.4	17.9	19.1
Aspartic acid	50.9	31.4	30.2
Cystine	6.4	2.0	1.7
Glutamic acid	56.1	17.6	19.9
Glycine	41.4	24.9	25.8
Proline	25.6	13.3	13.2
Serine	31.6	24.9	25.6
Tyrosine	15.4	9.2	9.2

**Table 3:** Apparent ileal digestibility of crude protein and amino acids in whole yeast and autolyzed yeast (%)^1)^

Item	Soybean meal	Whole yeast	Autolyzed yeast	SEM	p-value
Crude protein	75.47^b^	46.26^a^	68.09^b^	3.93	<0.001
Indispensable amino acids					
Arginine	86.53^b^	55.33^a^	85.12^b^	2.37	0.001
Histidine	86.64^c^	50.06^a^	78.66^b^	2.72	<0.001
Isoleucine	79.67^b^	51.19^a^	82.35^b^	3.53	0.002
Leucine	81.10^b^	50.24^a^	84.68^b^	3.08	<0.001
Lysine	85.51^b^	48.35^a^	86.10^b^	2.77	<0.001
Methionine	81.17^b^	45.74^a^	80.51^b^	3.27	<0.001
Phenylalanine	81.03^b^	48.89^a^	83.40^b^	3.20	<0.001
Threonine	75.74^c^	40.32^a^	67.89^b^	3.75	0.001
Valine	77.75^b^	50.28^a^	80.08^c^	4.00	0.003
Dispensable amino acids					
Alanine	74.94^b^	53.96^a^	83.52^c^	4.33	0.005
Aspartic acid	75.20^b^	45.42^a^	74.53^b^	4.38	0.007
Cystine	68.82^c^	29.22^a^	51.15^b^	7.39	0.008
Glutamic acid	74.87^b^	47.68^a^	81.78^b^	4.44	0.003
Glycine	61.93^b^	40.03^a^	67.58^b^	6.01	0.013
Proline	63.88^b^	31.07^a^	75.08^b^	5.27	<0.001
Serine	78.07^c^	43.48^a^	66.00^b^	3.80	<0.001
Tyrosine	77.74^b^	39.79^a^	78.73^b^	4.56	0.002

SEM, standard error of the means.

1) Each least squares mean represents 5 observations except for autolyzed yeast (6 observations).

a–c Within a row, least squares mean with a common superscript are not different at p<0.05.

**Table 4:** Standardized ileal digestibility (SID) of crude protein and amino acids in whole yeast and autolyzed yeast (%)^1),2)^

Item	Soybean meal	Whole yeast	Autolyzed yeast	SEM	p-value
Crude protein	82.47^b^	57.28^a^	78.64^b^	3.93	<0.001
Indispensable amino acids					
Arginine	89.75^b^	63.03^a^	93.50^b^	2.37	0.001
Histidine	88.02^b^	56.40^a^	84.86^b^	2.72	<0.001
Isoleucine	83.63^b^	55.84^a^	86.47^b^	3.53	0.002
Leucine	84.60^b^	55.24^a^	89.21^b^	3.08	<0.001
Lysine	90.49^b^	52.42^a^	89.70^b^	2.77	<0.001
Methionine	86.38^b^	51.17^a^	85.39^b^	3.27	<0.001
Phenylalanine	84.39^b^	54.04^a^	88.20^b^	3.20	<0.001
Threonine	83.05^c^	48.79^a^	75.77^b^	3.75	0.001
Valine	83.32^b^	54.76^a^	83.88^b^	4.00	0.003
Dispensable amino acids					
Alanine	80.60^b^	59.82^a^	88.52^c^	4.33	0.005
Aspartic acid	79.33^b^	50.90^a^	79.69^b^	4.38	0.007
Cystine	75.37^b^	42.28^a^	64.97^b^	7.39	0.017
Glutamic acid	78.39^b^	54.43^a^	87.19^b^	4.44	0.004
Glycine	75.86^b^	61.68^a^	86.97^c^	6.01	0.016
Proline	85.19^a^	74.43^a^	112.38^b^	5.27	0.002
Serine	83.44^c^	53.07^a^	75.88^b^	3.80	<0.001
Tyrosine	82.18^b^	45.26^a^	83.74^b^	4.56	0.002

SEM, standard error of the means; DM, dry matter.

1) Values for SID were calculated by correcting values for apparent ileal digestibility values for basal endogenous losses. Basal endogenous losses (g/kg of DM intake) were: crude protein, 12.34; arginine, 0.32; histidine, 0.06; isoleucine, 0.20; leucine, 0.36; lysine, 0.37; methionine, 0.09; phenylalanine, 0.21; threonine, 0.42; valine, 0.21; alanine, 0.34; aspartic acid, 0.57; cystine, 0.11; glutamic acid, 0.45; glycine, 1.80; proline, 1.73; serine, 0.72; tyrosine, 0.17.

2) Each least squares mean represents 5 observations except for autolyzed yeast (6 observations).

a–c Within a row, least squares mean with a common superscript are not different at p<0.05.
